# Physical Activity as a Key Factor in Elevated BMI Among Children with Developmental Coordination Disorder: A Cross-Sectional Analysis

**DOI:** 10.3390/children12091178

**Published:** 2025-09-04

**Authors:** Pablo Lizoain, Diana Rodriguez-Romero, Carmen Gándara, Leyre Gambra, Apolinar Varela, Nerea Crespo-Eguilaz, Sara Magallón, Martín Martínez

**Affiliations:** 1 Neurodevelopment and Learning Group, Education and Psychology Faculty, University of Navarra, 31009 Pamplona, Spain; drodriguezr@unav.es (D.R.-R.); cgandara@external.unav.es (C.G.); lgambra@unav.es (L.G.); agrana@unav.es (A.V.); necrespo@unav.es (N.C.-E.); smagallon@unav.es (S.M.); 2 Huarte de San Juan Center, 31003 Pamplona, Spain; 3 Pediatric Neurology Unit, Clinic University of Navarra, 31008 Pamplona, Spain; 4 Facultad de Educación, Universidad Internacional de La Rioja, 26006 Logroño, Spain; 5 Cognitive and Affective Methods in Psychology Group, Education and Psychology Faculty, University of Navarra, 31009 Pamplona, Spain; mmvillar@unav.es

**Keywords:** Development Coordination Disorder (DCD), motor impairment, physical activity, Body Mass Index (BMI), overweight, obesity, diet

## Abstract

**Highlights:**

**What are the main findings?**

- Children with Developmental Coordination Disorder (DCD) show lower levels of physical activity compared to typically developing peers.
- Despite similar dietary habits, children with DCD present higher BMI and body fat percentages, indicating increased obesity risk.

**What are the implications of the main findings?**

- Early identification and intervention in children with DCD are crucial to promote healthier lifestyles.
- Targeted strategies addressing physical inactivity may help reduce obesity risk and improve long-term health outcomes.

**Abstract:**

**Background/Objectives**: Developmental Coordination Disorder (DCD) affects approximately 5% of children globally and is characterized by motor difficulties that can negatively impact physical activity levels and increase the risk of obesity. Understanding the behavioral and physiological profiles of children with DCD is essential for early intervention. This cross-sectional study compared physical activity, dietary habits, and obesity indicators between children with DCD and their typically developing (TD) peers. **Methods**: A total of 243 children (134 boys, 109 girls) aged 6.65 to 11.65 years (M = 9.50, SD = 1.38) from two mainstream schools in the Chartered Community of Navarre, Spain, participated in the study. Children with conditions that could explain poor motor development were excluded based on DSM-5 Criterion D. Motor competence was evaluated using the FUNMOVES (n = 243) and MABC-2 tools (n = 49). Physical activity was assessed using the Physical Activity Questionnaire for Children (PAQ-C), dietary habits with the Krece Plus questionnaire, and obesity indicators through bioimpedance analysis (Body Mass Index and body fat percentage). Regression analyses were adjusted for age and sex. **Results**: Children classified with probable DCD (pDCD) using FUNMOVES showed significantly higher BMI (95% CI: 0.96 to 4.30 kg/m^2^) and body fat percentage (95% CI: 3.99 to 10.24%) than TD peers. Differences in physical activity were not statistically significant between DCD and TD (95% CI: −0.68 to 0.01. No significant differences were found in dietary quality. When assessing motor development with MABC-2, the results followed similar trends but lacked statistical significance. **Conclusions**: Children with DCD are at increased risk of overweight and obesity, primarily due to lower physical activity rather than dietary differences. Early identification and targeted interventions are essential to promote healthier lifestyles in this population.

## 1. Introduction

Developmental Coordination Disorder (DCD) is a neurodevelopmental disorder primarily characterized by difficulties in the execution of fundamental motor skills. These difficulties, present from the early stages of development, impact the daily lives of individuals with this disorder and cannot be explained by the presence of another condition [1]. With research indicating a rate of approximately 9% among school-aged children in Spain [2], the worldwide prevalence is estimated to range between 2% and 8% [1]. The co-occurrence of DCD with other learning and developmental disorders, such as Attention Deficit Hyperactivity Disorder (ADHD), dyslexia, language disorders, and autism spectrum disorders (ASDs), among others, has been demonstrated in various studies [3,4,5,6]. These comorbidities, along with the motor difficulties inherent to DCD, often lead to significant challenges in both academic performance and social interactions [7,8,9].

In recent years, research has revealed strong connections between mental and physical health problems, with excess weight being one of the most extensively studied topics across various fields [10]. Obesity has emerged as a prevalent and high-risk factor among individuals with certain neurodevelopmental and mental health disorders, including ADHD, ASD, DCD, and depression [11]. Individuals with DCD, in particular, often face multifaceted lifestyle challenges that extend beyond motor impairments. Difficulties in maintaining adequate levels of physical activity, regulating sedentary behavior, managing sleep patterns, and following balanced nutritional habits contribute to an overall unhealthy lifestyle [12].

Overweight and obesity are defined as abnormal or excessive fat accumulation that may compromise health [13]. The most widely used method for assessing these conditions is the Body Mass Index (BMI), calculated as weight in kilograms divided by height in meters squared (kg/m^2^). Based on this index, individuals are categorized as underweight, normal weight, overweight, or obese (see Appendix B). Four classification systems are commonly applied: the Orbegozo Foundation growth charts, the criteria proposed by the Spanish Association of Pediatrics (AEP), the World Health Organization (WHO) growth standards, and the Arriba classification system [14,15,16,17].

Although BMI is widely used, its validity as an indicator of adiposity has been questioned in the literature [18,19,20]. Due to these limitations, complementary methods for estimating body fat have been developed. These include anthropometric techniques, such as skinfold thickness measurements, and bioelectrical impedance analysis (BIA) [21]. While skinfold assessment relies on regression equations based on the number and location of folds, BIA offers a quicker, non-invasive alternative. It estimates total and visceral fat by measuring the body’s resistance to a low-voltage electrical current. BIA is often preferred over skinfold measurements in large-scale field-based investigations due to its practicality and time efficiency [22,23].

Children and adolescents who are overweight are at greater risk of developing obesity in adulthood [24]. This significantly increases the likelihood of chronic diseases such as diabetes, hypertension, and cardiovascular problems [25]. In the case of individuals with DCD, this condition is particularly relevant, as they can be considered a population at risk. This hypothesis is supported by a meta-analysis [26], which found that children with DCD have 1.87 times higher odds of being overweight or obese than their TD peers (95% CI = 1.43–2.44) [26].

Physical activity has been consistently identified as a critical factor in preventing and managing overweight and obesity, with higher activity levels correlating with a reduced risk of these conditions [27,28]. However, children with DCD typically engage in lower levels of moderate-to-vigorous physical activity and spend more time in sedentary behaviors than their typically developing peers. A recent systematic review and meta-analysis confirmed that school-aged children with DCD (6–14 years) participate significantly less in moderate-to-vigorous physical activity (standardized mean difference = −0.27, 95% CI: −0.38 to −0.16), underscoring the need for increased awareness among parents and healthcare providers [29,30]. The motor impairments inherent to DCD often result in avoidance or reduced participation in coordinated physical activities [3,31,32,33]. This issue is exacerbated in social or group settings, potentially initiating a negative feedback loop where limited engagement hampers motor skill development, which in turn diminishes physical fitness and contributes to weight gain. Importantly, this lack of physical activity appears to be a characteristic more specific to children with DCD, distinguishing them from their peers.

Dietary habits also play a crucial role in the development and maintenance of overweight and obesity. While diet problems are likely more widespread among all children, it remains essential to understand whether differences in dietary patterns exist between children with DCD and typically developing peers [34,35]. Clarifying this distinction will help identify the most effective intervention targets and whether elevated obesity rates in the DCD population should be addressed primarily through nutritional programs or if the focus should remain on physical activity.

Primary (confirmatory) hypothesis. Children with pDCD will have significantly higher adiposity than TD peers, operationalized as higher BMI and body fat percentage. Secondary (exploratory) hypothesis. Children with pDCD will exhibit lower physical activity levels than TD peers.

Therefore, the present study aims to evaluate differences in physical activity, body composition, and dietary patterns between children with DCD and their typically developing peers. We further hypothesize that lower physical activity levels, which are more characteristic of DCD, alongside potentially poorer dietary habits common to many children, contribute jointly to the increased risk of overweight and obesity in this population. This approach will help determine whether interventions should prioritize physical activity, diet, or an integrated strategy to reduce obesity risk in children with DCD.

## 2. Materials and Methods

### 2.1. Study Population

**Figure 1:**
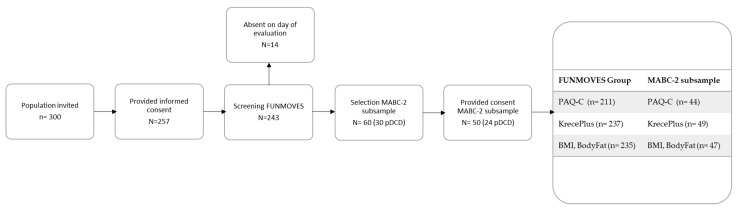
Flowchart detailing the inclusion and exclusion of participants throughout the study.

This study involved primary school children from two mainstream educational centers located in the Chartered Community of Navarre (Spain) (Figure 1). All students from grades 1 to 6 were invited to participate. Children with any condition that could be considered the primary cause of poor motor development were excluded, in accordance with DCD Criterion D of the DSM-5. Excluded conditions included, among others, intellectual disability, visual impairment, and pervasive developmental disorders.

The study sample comprised a total of 243 participants (134 boys and 109 girls) aged between 6.65 and 11.65 years (M = 9.50, SD = 1.38). Participants were distributed across six consecutive school years, with varying proportions of boys and girls per year. The mean body fat percentage was 21.72% (SD = 7.67), while the mean Body Mass Index (BMI) was 17.79 kg/m^2^ (SD = 3.08). Detailed descriptive statistics by academic year, including sex distribution, age, body fat, and BMI, are presented in Table 1.

Data were collected from children whose parents or legal guardians provided written informed consent authorizing their participation in the first stage of the study, conducted between 30 January and 31 March 2023. For those involved in the second stage of the study, carried out between 25 October and 23 November 2023, an additional written informed consent was required, also signed by the same legal guardians. All consent forms were duly signed and securely stored. The study received approval from the Ethics Committee of the University of Navarra (reference: 2022.088). No waiver of consent was granted.

### 2.2. Study Design

First, two questionnaires were completed to assess physical activity levels and diet quality. For students in grades 1 to 3 of primary education, these questionnaires were completed by parents or legal guardians. Once the questionnaires were submitted, an anthropometric assessment was conducted, collecting data on weight, height, and body fat percentage for each participant. Subsequently, a screening evaluation using a validated tool for the screening of motor impairment was implemented by the schools. Finally, those identified as being at risk of motor difficulties, along with a control group matched by school grade, underwent assessment using the gold-standard tool for the assessment of motor skills. No a priori sample size calculation was conducted because all available students were included (census-like design). Reporting adhered to the STROBE checklist for cross-sectional observational studies [36].

### 2.3. Tools

#### 2.3.1. Lifestyle Questionnaires

Lifestyle was assessed using two questionnaires validated for the Spanish population. The Krece Plus questionnaire [37] evaluates dietary habits and physical activity in children and adolescents, providing both a total score and a categorical classification (poor, needs improvement, optimal) of dietary quality. However, the physical activity component of Krece Plus was not used, as a more comprehensive instrument was available for this purpose. Physical activity was assessed using the Physical Activity Questionnaire for Children (PAQ-C), which measures general activity levels over the previous week and includes a specific item assessing typical weekly activity [38]. Categorical classifications were derived from both the overall PAQ-C score and the weekly activity item. Participants who were unable to engage in regular physical activity due to illness or injury were excluded from the analyses. Full versions of both questionnaires are provided in Appendix C (Krece Plus) and Appendix D (PAQ-C).

#### 2.3.2. Anthropometric Data Collection

Anthropometric data collection included three variables: height, weight, and body fat percentage. Based on the height and weight measurements, the Body Mass Index (BMI) was calculated for each participant to provide an additional indicator of body composition. Height was measured using a standard measuring tape, while weight and body fat percentage were obtained using a bioelectrical impedance scale: Tanita RD-545, (Tanita Corporation, Tokyo, Japan), which offers a precision of 50 g. The device provides full-body fat percentage estimates for each participant. All measurements were recorded digitally and exported in “.csv” format via the scale’s proprietary application. Each participant’s data were matched using a unique identification code, along with the corresponding date and time of measurement, to ensure accurate data management and traceability.

#### 2.3.3. Screening of Fundamental Movement Skills with FUNMOVES

The Spanish version of the FUNMOVES instrument was employed to assess fundamental motor skills in school-aged children. FUNMOVES (this article is currently under review in *PLOS ONE* (date of submission: 4 July 2025), and the Spanish adaptation of FUNMOVES preserves the psychometric properties and structure of the original tool) is a school-based screening tool designed to detect motor coordination challenges through a structured set of physical tasks. It evaluates key aspects of motor competence, such as balance, coordination, and object control, using activities that are feasible within physical education classes. The tool is time-efficient, requires only basic equipment, and allows for standardized scoring based on age-stratified normative data. Published work demonstrates the validity/reliability of FUNMOVES in school contexts, including Rasch-based development and subsequent validation in practice [39,40].

#### 2.3.4. Assessment of Fundamental Movement Skills with Movement Assessment Battery for Children—Second Edition (MABC-2)

A subsample of participants (n = 49) was assessed using the most recent version of the Movement Assessment Battery for Children—Second Edition (MABC-2; Pearson Assessment, Madrid, Spain), validated for the Spanish population. Consistent with international recommendations, MABC-2 served as the gold standard for motor competence; in the subsample, pDCD was standardized as MABC-2 total percentile < 15th [41]. It consists of a series of structured tasks grouped into three subscales, manual dexterity, aiming and catching, and balance, which together yield a total motor score. The full assessment takes approximately 40 min per child and provides age-normed scores that allow for the identification and quantification of motor impairments, offering a comprehensive evaluation of motor competence [42]. This operationalizes DSM.5 Criterion A (motor difficulties). While Criterion D was addressed by excluding conditions that could explain poor motor development, Criteria B and C were not systematically assessed; therefore, we refer to pDCD rather than a clinical diagnosis.

FUNMOVES captures everyday motor performance in naturalistic school settings, providing insight into children’s functional motor competence in their daily environment. In contrast, MABC-2 offers a standardized assessment suitable for clinical decision-making. Together, these tools have complementary utility, allowing for both ecological validity and clinical applicability. The use of FUNMOVES in schools is feasible, with the manual freely available online, and it meets 5 out of 7 criteria for school-based motor assessments established by Klingberg et al. [43]. A summary comparison of the main features, strengths, limitations, and utility of both tools is provided in Table 2.

Accordingly, all analyses are reported separately for the FUNMOVES screening status (full sample) and the MABC-2 gold-standard status (subsample).

### 2.4. Procedure

This study employed a cross-sectional design with purposive sampling. Initially, data were filtered to retain only valid records for each variable. Participants were included if they had completed the fundamental motor skills assessment with FUNMOVES. Individuals with missing data in any lifestyle-related variables, PAQ-C, Krece Plus, or anthropometric measures, were excluded from the corresponding analyses.

Lifestyle assessments were conducted concurrently with the administration of FUNMOVES. These assessments included the PAQ-C questionnaire, the Krece Plus test, and anthropometric measurements. Additionally, the PAQ-C item measuring weekly physical activity was extracted and used as a separate classification variable.

As part of the procedure, a new composite variable was generated to classify Body Mass Index (BMI). Four reference systems were used: Orbegozo Foundation, Spanish Pediatric Association (Asociación Española de Pediatría—AEP), World Health Organization (WHO), and the classification proposed by Arriba. Each system provided specific thresholds for categorizing participants as underweight, normal weight, overweight, or obese (see Appendix A, Table A1, Table A2, Table A3 and Table A4). A majority vote approach was applied to determine the final classification. In cases of a tie (2 vs. 2), the AEP system was used as a tiebreaker due to its high concordance with the other references.

Screening stage (FUNMOVES). Children were initially screened in schools using the FUNMOVES assessment. Those scoring below the 10th percentile for their age, or below the 15th percentile in combination with a flag from the physical education teacher indicating potential motor difficulties, were classified as probable Developmental Coordination Disorder (pDCD). The remaining participants were categorized as typically developing (TD).

Gold-standard confirmation (MABC-2 subsample). A subsample comprising children identified as pDCD and a matched group of TD peers subsequently completed the MABC-2 assessment. Within this subsample, final group status was standardized based on the MABC-2 total percentile. Children scoring below the 15th percentile were assigned to the pDCD group, while the remaining participants were classified as TD. No significant age differences were observed between the groups.

### 2.5. Data Analysis

All statistical analyses were performed in R (version 4.3.0).

Descriptive statistics were calculated for all variables of interest. Continuous variables were summarized using means and standard deviations, while categorical variables were expressed as frequencies and percentages. Comparisons between TD and those at risk of Developmental Coordination Disorder (pDCD) were conducted separately based on two classification criteria: (a) the FUNMOVES screening status in the full sample and (b) the MABC-2 gold-standard status in the subsample.

To assess group differences in diet, physical activity (PA), body fat percentage, and BMI, linear regression models were fitted with motor competence group (TD vs. pDCD) as the primary predictor. All models were adjusted for age and sex. For robustness, analyses were conducted using both raw scores and z-scores (standardized using the total sample standard deviation). The 95% confidence intervals of the estimated group differences were obtained via nonparametric bootstrapping with 5000 resamples, using the boot package. We obtained nonparametric bootstrap 95% CIs (5000 resamples) for all estimates. No additional multiplicity adjustment was applied across exploratory outcomes; these are reported with exact estimates and CIs and interpreted cautiously. A group difference was considered statistically significant when the confidence interval did not include zero, as this indicates that the estimated effect is consistently either positive or negative [44].

In a secondary analysis, the unique contribution of motor competence status to BMI was examined by fitting a series of hierarchical regression models. These models progressively adjusted for potential behavioral confounders, namely, diet quality (measured via the Krece Plus total score) and physical activity levels (measured using the PAQ-C, including both total and weekly scores). The standardized effect of motor status was calculated by dividing the estimated coefficient by the sample-wide BMI standard deviation (SD = 3.08), and the results were expressed as z-scores. These analyses were conducted using the broom and dplyr packages for model tidying and data manipulation [45,46].

Finally, mediation analyses were conducted to explore whether physical activity and diet quality mediated the relationship between motor competence status (TD vs. pDCD) and BMI. Separate models were fitted for each potential mediator: weekly physical activity (PAQ-C weekly score), total physical activity (PAQ-C total score), and diet quality (Krece Plus total score). For each analysis, a linear regression model was first fitted to estimate the effect of motor competence status on the mediator, followed by a second model estimating the effect of both motor competence status and the mediator on BMI. Mediation effects were assessed using the mediate function from the mediation R package(version 4.5.1), with nonparametric bootstrapping (5000 resamples) to obtain 95% confidence intervals for the indirect effects [44]. Given the cross-sectional design, these analyses are considered exploratory and hypothesis-generating, and no causal inferences should be drawn [47,48].

Multiplicity policy. We pre-specified BMI and body fat as primary endpoints and considered diet and physical activity (total and weekly) as secondary, exploratory outcomes. Confirmatory inference was restricted to the primary endpoints. For all models, we report nonparametric bootstrap 95% CIs (5000 resamples). We did not apply a formal multiplicity correction across exploratory outcomes; these results are presented for estimation and hypothesis generation and are interpreted cautiously.

For primary endpoints (BMI, body fat), statistical significance was defined as a 95% CI, excluding zero. For mediation models, two-sided *p*-values are reported with α = 0.05.

### 2.6. Generative Artificial Intelligence (genAI)

In the preparation of this manuscript, generative artificial intelligence (GenAI) tools were employed to support specific aspects of the research process. AI-based translation tools were used to assist in the accurate and context-sensitive translation of content between Spanish and English. Additionally, GenAI was utilized for the generation, revision, and optimization of R code, which was instrumental in conducting the statistical analyses presented in this study. These applications of GenAI were limited to technical and linguistic support and did not influence the interpretation of results or the formulation of scientific conclusions.

## 3. Results

The following results pertain to participants’ diet, physical activity, and body composition. In some cases, participants completed only certain parts of the study; therefore, the total sample size varies across the different sections.

### 3.1. Diet

A total of 237 children completed the Krece Plus questionnaire and participated in the FUNMOVES assessment, of which 208 were TD, representing 87.6% of the sample (Table A1). Within this group, 49 participants also underwent evaluation using MABC-2. The overall mean diet quality score for the full sample was 6.95 (SD = 1.99). Importantly, the majority of participants (61.2%) did not meet the criteria for an optimal diet, being classified within the “Very low quality” or “Needs improvement” categories. Diet quality classifications for both assessment tools are summarized in Table A1. No significant differences were observed between the TD and pDCD groups based on either the FUNMOVES or MABC-2 classification systems.

### 3.2. Physical Activity

Physical activity (PA) levels were assessed using the Physical Activity Questionnaire for Children (PAQ-C), completed by 211 participants. Based on the total PAQ-C score, 107 children were classified as having low or very low physical activity levels. When considering the weekly PAQ-C score, 76 children failed to meet the minimum recommended levels of physical activity.

Table A2 and Table A3 present the distribution of PA classifications for both total and weekly scores, respectively. According to the FUNMOVES assessment, a larger proportion of children in the pDCD group exhibited very low and low PA levels compared to their TD peers. Similar trends were observed in the subsample assessed with MABC-2. The full breakdown of PA categories by group is summarized in the tables below.

In addition to categorical classifications, the raw PAQ-C scores were analyzed to provide a continuous measure of physical activity levels. Table A4 summarizes the mean and standard deviation of total and weekly PAQ-C scores for both the TD and pDCD groups. Across both assessment tools, FUNMOVES and MABC-2, children in the TD group consistently exhibited higher mean scores than their pDCD counterparts, indicating greater overall physical activity.

### 3.3. Body Fat

Descriptive statistics for body fat percentage are presented in Table A5. Children identified as at risk for DCD (pDCD) showed higher mean body fat percentages compared to their TD peers across both assessment groups. In the sample assessed with FUNMOVES, the mean body fat percentage was 28.9% (SD = 8.69) for the pDCD group and 22.06% (SD = 5.81) for the TD group. In those assessed with MABC-2, the pDCD group had a mean of 28.46% (SD = 10.99), while the TD group had a mean of 24.82% (SD = 6.40).

These results suggest a consistent trend of higher adiposity in children at risk for DCD, regardless of the motor assessment tool used.

### 3.4. Body Mass Index

**Figure 2:**
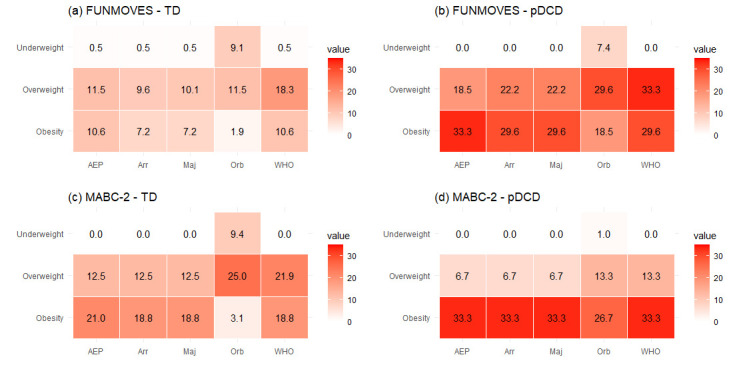
Heat maps showing the distribution of values across different weight categories (underweight, overweight, obesity) and assessment types (AEP, Orbegozo, WHO, Arriba, majority), each corresponding to a different classification group: (**a**) TD classified with FUNMOVES; (**b**) pDCD classified with FUNMOVES; (**c**) TD classified with MABC-2; (**d**) pDCD classified with MABC-2.

The relationship between motor competence and nutritional status was examined using five BMI classification systems: AEP, Fundación Orbegozo, WHO, Arriba2016, and a majority vote approach. Participants were classified into TD and pDCD groups based on both the FUNMOVES (n = 208 TD; n = 27 pDCD) and MABC-2 assessments (n = 32 TD; n = 15 pDCD). When motor status was defined by FUNMOVES, consistent and statistically significant differences emerged across all BMI classification systems, with the pDCD group showing a markedly higher prevalence of overweight and obesity. For instance, obesity rates in the pDCD group ranged from 18.5% to 33.3%, compared to 1.9% to 10.6% in the TD group, depending on the classification system (Figure 2).

### 3.5. Post Hoc Sensitivity/Power

A post hoc sensitivity analysis was conducted to assess the study’s power to detect differences in the primary outcomes. For the FUNMOVES group (TD = 208; pDCD = 27), the study had 80% power to detect an effect size of d ≥ 0.57. For the MABC-2 subsample (TD = 32; pDCD = 15), 80% power was achieved for d ≥ 0.88. These calculations were based on a normal approximation for two-tailed tests with α = 0.05. The results suggest that non-significant contrasts in the MABC-2 subsample may reflect limited statistical power rather than the absence of effects [49].

### 3.6. Differences Between the TD and pDCD Groups

Table 3A presents the adjusted differences between the TD and pDCD groups using raw scores, with all models adjusted for age and sex. These raw scores are expressed in the original units of each variable, allowing for a direct interpretation of the magnitude of the differences. No significant differences were observed in diet quality scores across groups, as the 95% confidence intervals included zero for both the FUNMOVES and MABC-2 classifications. In terms of physical activity in the FUNMOVES classification, the total adjusted scores also included zero, with a confidence interval ranging from –0.68 to 0.01. This suggests that children in the pDCD group may engage in fewer weekly activity units compared to their TD peers. The most pronounced differences were observed in adiposity indicators. When classified using FUNMOVES, children in the pDCD group exhibited significantly higher body fat percentages and BMI values. Specifically, the adjusted difference in body fat ranged from 3.99% to 10.24%, and BMI differences ranged from 0.96 to 4.30 kg/m^2^. These confidence intervals did not include zero, indicating statistically significant group differences. In contrast, although the MABC-2 classification showed similar directional trends in adiposity, the confidence intervals for both body fat and BMI included zero, and thus the differences were not statistically significant.

To facilitate comparisons across variables with different units and scales, Table 3B presents the same group differences using standardized z-scores. These scores express differences in terms of standard deviations, providing a clearer understanding of the relative magnitude of effects. As with the raw scores, no significant differences were observed in diet quality. For the FUNMOVES classification, the weekly PA difference was borderline (95% CI = −0.88 to 0.01) and is interpreted as exploratory per our multiplicity policy. Regarding adiposity, the standardized results confirmed the findings from raw scores. Children in the pDCD group classified by FUNMOVES had significantly higher z-scores for both body fat and BMI, with confidence intervals ranging from 0.61 to 1.55 and from 0.31 to 1.40, respectively. These values indicate moderate to large effect sizes. In contrast, the MABC-2 classification did not yield statistically significant differences in any of the standardized variables, although the direction of the effects remained consistent with those observed using FUNMOVES. This pattern suggests that while both classification tools point toward similar trends, the FUNMOVES classification is more sensitive in detecting group differences, particularly in adiposity-related outcomes.

**Figure 3:**
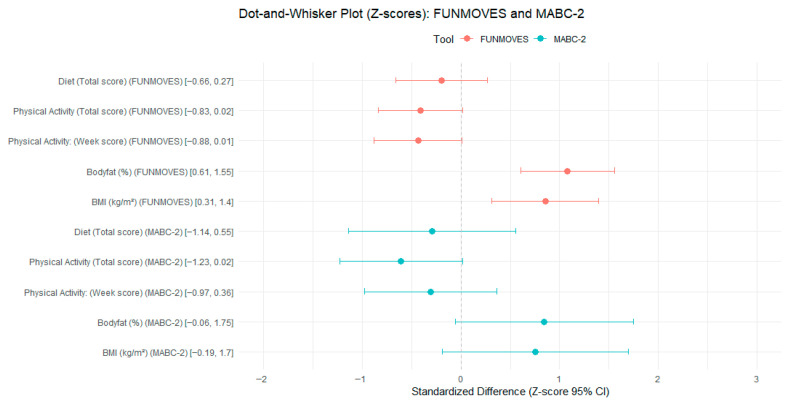
Standardized group differences (z-scores) in diet, physical activity, BMI, and body fat according to FUNMOVES and MABC-2 classifications.

For the group classified using MABC-2, no significant differences between the TD and pDCD groups were observed in any of the evaluated variables, as all confidence intervals included zero. However, it is worth noting that the direction of the results appears to follow a similar trend to that observed with the FUNMOVES classification, particularly in physical activity measures, suggesting a potential underlying pattern that may not have reached statistical significance but aligns with the tendencies identified using FUNMOVES. This pattern is visually represented in Figure 3, which illustrates the parallel trends in group differences across both classification tools.

To further examine the relationship between motor competence and BMI, a series of regression models was conducted using the FUNMOVES classification as the main predictor, progressively adjusting for diet quality and physical activity. The standardized coefficients (z-scores) of the motor status variable across models ranged from 0.765 to 0.821, indicating a stable and moderate association between lower motor competence and higher BMI.

The base model, which did not include behavioral covariates, yielded a z-score of 0.821. Adjusting for diet quality slightly reduced this estimate (z = 0.808), while the inclusion of total or weekly physical activity scores further attenuated the association (z = 0.786 and z = 0.767, respectively). Models that simultaneously accounted for both diet and physical activity produced similar estimates (z = 0.788 with total PA; z = 0.765 with weekly PA), suggesting that the observed relationship remains relatively consistent and is not substantially diminished by these covariates. All standardized effects are visually summarized in Figure 3, which displays the z-scores across the different models.

Mediation analyses were conducted to examine whether the effects of motor competence, measured through FUNMOVES status and percentiles, as well as total and subscale percentiles from the MABC-2 battery, on Body Mass Index (BMI) were mediated by dietary habits, weekly physical activity, and total physical activity.

The percentile score in FUNMOVES showed significant total effects on BMI (total effect = −0.0245, *p* < 0.001), with partial mediation through both weekly (Prop. Mediated = 12.9%, *p* = 0.0184) and total physical activity (Prop. Mediated = 11.1%, *p* = 0.0396), suggesting that physical activity plays a meaningful role in the relationship between motor competence and BMI. The FUNMOVES status also demonstrated partial mediation through physical activity, though with a smaller proportion mediated (Prop. Mediated = 11.8%, *p* = 0.0308 for weekly physical activity).

**Figure 4:**
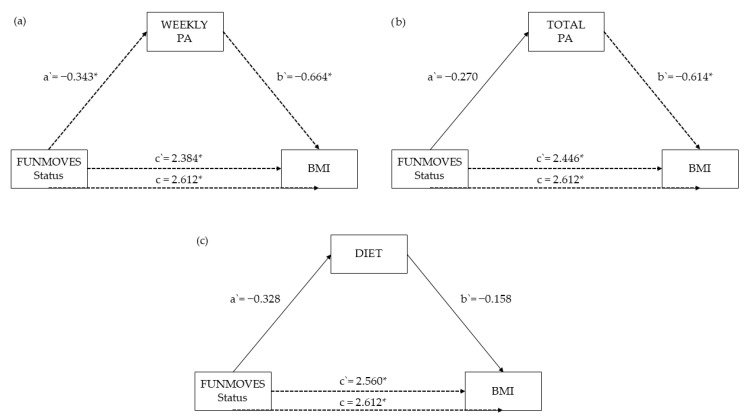
Path coefficients (a′, b′, c′, c) from mediation models relating motor competence (FUNMOVES status) to BMI via (**a**) total physical activity, (**b**) weekly physical activity, and (**c**) diet. a′ indicates the effect of the predictor on the mediator, b′ is the effect of the mediator on BMI, c′ is the direct effect, and c is the total effect. * indicates statistically significant path (*p* < 0.05).

Figure 4 displays the path coefficients from mediation models examining the role of physical activity (weekly and total) and dietary habits as potential mediators in the relationship between FUNMOVES status and Body Mass Index (BMI). Across all three models, the direct effect of FUNMOVES status on BMI (c′) remained statistically significant: c′ = 2.384, *p* = 0.007 for weekly physical activity (Figure 4a), c′ = 2.446, *p* = 0.004 for total physical activity (Figure 4b), and c′ = 2.560, *p* = 0.005 for diet (Figure 4c). These results indicate that having low motor competence (i.e., being classified as FUNMOVES) is directly associated with higher BMI, regardless of the mediator considered.

Both weekly and total physical activity significantly mediated this relationship. In the weekly physical activity model (Figure 4a), FUNMOVES status had a significant negative effect on weekly PA (a′ = −0.343, *p* = 0.034), and weekly PA in turn showed a significant negative association with BMI (b′ = −0.664, *p* = 0.011). This suggests that lower motor competence leads to reduced physical activity, which is associated with higher BMI.

A similar but slightly weaker pattern emerged in the total physical activity model (Figure 4b), where a′ = −0.270, *p* = 0.051 and b′ = −0.614, *p* = 0.044, again supporting the mediating role of physical activity in the link between motor competence and BMI, though with a marginally non-significant a′ path.

In contrast, the diet model (Figure 4c) did not support a significant mediation effect. Although the direct effect remained significant (c′ = 2.560, *p* = 0.005), neither the path from FUNMOVES status to diet (a′ = −0.328, *p* = 0.429) nor the path from diet to BMI (b′ = −0.158, *p* = 0.118) reached statistical significance. This suggests that dietary habits do not meaningfully mediate the association between motor competence and BMI in this sample.

Taken together, these findings underscore the importance of physical activity, especially weekly physical activity, as a significant mechanism linking motor competence and BMI, justifying its inclusion in further mediation analyses.

The total percentile score in MABC-2 and the balance subscale percentile showed significant direct effects on BMI (e.g., MABC total: ADE = −0.0447, *p* = 0.0396; balance: ADE = −0.0532, *p* = 0.0084), but there was no significant mediation through diet or physical activity (all *p* > 0.5 for ACME and Prop. Mediated). The manual dexterity and aiming and catching subscales did not show significant direct or mediated effects (e.g., MABC_MD: total effect = −0.0082, *p* = 0.7236; MABC_AC: total effect = 0.0046, *p* = 0.8408). Similarly, the status based on the total MABC-2 score showed no significant direct or mediated effects on BMI (total effect = 1.43, *p* = 0.3432). The full numerical results for each path coefficient and mediation estimate are provided in Appendix D.

## 4. Discussion

This study examined the relationship between motor competence and health-related outcomes in school-aged children, including diet quality, physical activity (PA), body composition, and BMI. Differences were explored using two motor assessment tools, FUNMOVES and MABC-2, while adjusting for age and sex.

The findings revealed no significant differences in diet quality between children classified as TD and those identified as pDCD, regardless of the motor assessment tool used. Although mean diet scores tended to be lower in the pDCD group, reduced motor competence was not robustly associated with poorer dietary patterns in this sample.

In contrast, differences in physical activity levels were more pronounced. Children identified as pDCD demonstrated consistently lower PAQ-C scores compared to their TD peers, particularly when classified using FUNMOVES, a finding that aligns with previous research highlighting reduced physical activity participation among children with motor coordination difficulties [50,51,52]. The estimates approached statistical significance and were directionally consistent, suggesting a meaningful difference in physical activity behavior between groups. This tendency was observed for both the total and weekly PA measures. Comparisons based on MABC-2 classifications followed a similar, although slightly attenuated, pattern.

The body composition results further supported the existence of health disparities associated with motor competence. Children classified as pDCD using FUNMOVES exhibited significantly higher body fat percentages and BMI values, even after adjusting for age and sex, which is consistent with prior research [26,53]. These associations remained moderate to strong when expressed as standardized effect sizes. Conversely, the MABC-2-based comparisons displayed the same directional trend but with wider confidence intervals that encompassed zero, reflecting greater statistical uncertainty due to the smaller sample size.

To better understand the mechanisms underlying the elevated BMI observed in children with probable DCD, we first examined whether this association was more strongly influenced by diet quality or physical activity. By progressively adjusting for these variables in separate regression models, we observed that the standardized effect of motor status on BMI remained largely unchanged after accounting for diet quality. However, when adjusting for physical activity, particularly weekly activity levels, a more noticeable attenuation in the strength of the association was observed. This pattern suggests that physical activity may play a more influential role than diet in explaining the higher BMI and increased prevalence of overweight and obesity among children with reduced motor competence [54,55,56,57].

Building on these findings, formal mediation analyses further clarified these relationships. When using FUNMOVES as the motor competence indicator, physical activity was found to partially mediate the association with BMI, with both weekly and total PA showing statistically significant indirect effects. Notably, up to 13% of the total effect of FUNMOVES percentiles on BMI was explained by weekly physical activity, reinforcing the interpretation that PA serves as a key behavioral pathway linking motor competence to body composition. In contrast, diet quality did not significantly mediate these associations.

While diet quality did not significantly mediate the associations, this finding should be interpreted with caution. Methodological factors may have contributed, such as reliance on self-reported dietary data and the limited sensitivity of the Krece Plus questionnaire to capture subtle variations in diet quality. At the same time, it is also plausible that diet quality is similarly suboptimal across children with and without motor difficulties, which would reduce its potential explanatory power in this context. Moreover, to date, the literature has not identified diet as a specific concern among children with DCD when compared to their typically developing peers, and the present results are consistent with this absence of evidence. Together, these considerations may help to explain the lack of a discernible mediating effect of diet.

Interestingly, MABC-2-based models revealed a different pattern: while some subscales, such as balance, exhibited direct associations with BMI, there was no evidence of mediation through either physical activity or dietary habits [58]. This suggests that the relationship between motor competence and BMI may depend not only on the assessment method but also on the specific constructs captured by each tool. FUNMOVES may better reflect everyday motor performance in school contexts, where physical activity behaviors are expressed, whereas MABC-2 may capture more isolated motor capacities with weaker behavioral connections. Given its higher ecological validity in school settings, FUNMOVES provides a unique opportunity for the early identification of children with motor difficulties. Its use in everyday educational contexts allows for the timely detection and monitoring of functional motor competence. Consequently, implementing FUNMOVES in schools could support targeted interventions and promote better long-term outcomes.

These findings carry important implications for intervention strategies. While promoting healthy dietary habits remains essential, our results point to physical activity as a more relevant and potentially modifiable target for preventing excessive weight gain in children with motor difficulties. Enhancing opportunities for accessible, inclusive, and enjoyable physical activity may be key to breaking the cycle between low motor competence and poor health outcomes in this vulnerable population. Furthermore, the observed differences between assessment tools underscore the importance of selecting appropriate measures of motor competence that align with intervention goals and ecological validity. Systematic screening and early identification of children with DCD can guide public school policies and support the implementation of adapted motor programs within the curriculum. Such programs may improve motor skills, encourage physical activity, and mitigate negative health outcomes, ultimately facilitating evidence-based decision-making at both the school and district levels. At scale, universal screening with FUNMOVES and targeted MABC-2 referrals can underpin tiered school policies for early support and adapted PE programming.

## 5. Conclusions

This study identifies an increased risk of obesity in children with pDCD, highlighting a critical area of concern for their overall health and development. The observed differences in adiposity appear to be more strongly associated with reduced participation in physical activity than with dietary quality. This suggests that motor difficulties may act as a barrier to active engagement, which in turn contributes to higher BMI levels in this population.

Given these findings, it is essential to prioritize strategies that promote participation in physical activity among children with motor challenges. Interventions should not only aim to improve motor competence but also foster inclusive environments that encourage sustained and meaningful engagement in movement-based activities. Understanding and addressing these participation barriers may be key to reducing obesity risk and supporting healthier developmental trajectories.

## 6. Limitations and Future Proposals

This study presents several limitations that should be considered. First, the relatively small sample size may limit the generalizability of the findings. Although both a screening tool and the gold-standard MABC-2 were used, only a subsample of children identified as at risk for DCD, along with a control group, were assessed using MABC-2. Time constraints prevented the assessment of the entire screened population with the gold-standard instrument.

Second, physical activity levels were measured using self-reported questionnaires. This method may introduce reporting bias and reduce data accuracy. Future research should incorporate objective tools, such as accelerometers, to improve measurement reliability.

Third, although this study examined obesity risk in children with DCD, further research is needed to assess the appropriateness of obesity risk screening in this population. Early identification of relevant indicators may support the development of targeted preventive strategies.

Given the cross-sectional design, the directionality of associations cannot be established, and the possibility of reverse causality (e.g., higher body weight leading to lower physical activity rather than the opposite) cannot be ruled out.

Future interventions should explore diverse approaches to increasing physical activity. These may include minimizing exposure to situations where motor difficulties are highly visible but also promoting participation in inclusive, socially meaningful contexts. School-based programs that emphasize enjoyment, cooperation, and individual strengths could be particularly effective.

Larger and more diverse samples are also needed in future studies. This would enhance the robustness of the findings and allow for subgroup analyses based on age, sex, or severity of motor impairment.

## Figures and Tables

**Table 1:** Demographic description of participants.

	Sex		Age (Years)		Body Fat		BMI	
Year	Boys (%)	Girls	Mean	SD	Mean	SD	Mean	SD
1	8 (66.67)	4	6.65	0.30	22.88	4.13	16.59	2.63
2	13 (59.09)	9	7.67	0.35	25.1	9.22	18.13	4.18
3	48 (53.93)	41	8.69	0.29	21.77	5.84	16.93	2.55
4	5 (33.33)	10	9.72	0.42	27.19	8.43	19.89	3.47
5	47 (54.02)	40	10.70	0.31	19.09	8.59	18.12	2.92
6	13 (72.22)	5	11.65	0.35	23.78	6.57	19.19	3.34
Total	134 (56.54)	109	9.50	1.38	21.72	7.67	17.79	3.08

**Table 2:** Differences between FUNMOVES and MABC-2.

Variable	FUNMOVES	MABC
Setting context	School-based screening	Gold standard
Number of items	6 (running, jumping, hopping, throwing, kicking, and balance)	10–11 (3 subscales)
Duration	60 min—30 children	40 min per child
Complexity	Low/moderate	Moderate/high
Outcome	Age-normed screening score	3 subscales (manual dexterity, aiming and catching, and balance) and total score
Cutoffs	Percentiles: <10th/<15th with teacher flag pDCD	Percentiles: <5th DCD; <15th pDCD
Psychometrics	Recent validation in school settings	Standardized manual
Strengths	Ecological validity, free	Diagnostic rigor
Limitations	Not diagnostic, local norms	Equipment, duration, and training for assessing

**Table 3:** (**A**) Differences between groups adjusted by sex and age. (**B**) Standardized (z-score) differences between the TD and pDCD groups adjusted for sex and age.

**(A) Raw Score**		
**Tool**	**Variable**	**95%CI**
FUNMOVES	Diet (total score)	−1.11 to 0.45
	PA (total score)	−0.55 to 0.01
	PA (weekly score)	−0.68 to 0.01
	Body fat (%)	3.99 to 10.24 *
	BMI (kg/m^2^)	0.96 to 4.3 *
MABC-2	Diet (total score)	−1.91 to 0.93
	PA (total score)	−0.81 to 0.01
	PA: (weekly score)	−0.75 to 0.28
	Body fat (%)	−0.39 to 11.52
	BMI (kg/m^2^)	−0.59 to 5.23
**(B) z-Score**		
**Tool**	**Variable**	**95%CI**
FUNMOVES	Diet (total score)	−0.66 to 0.27
	PA (total score)	−0.83 to 0.02
	PA (weekly score)	−0.88 to 0.01
	Body fat (%)	0.61 to 1.55 *
	BMI (kg/m^2^)	0.31 to 1.40 *
MABC-2	Diet (total score)	−1.13 to 0.55
	PA (total score)	−1.23 to 0.01
	PA: (weekly score)	−0.97 to 0.36
	Body fat (%)	−0.05 to 1.75
	BMI (kg/m^2^)	−0.19 to 1.69

Note: An asterisk (*) denotes statistical significance, inferred from the 95% confidence interval not including zero.

**Figure A1:**
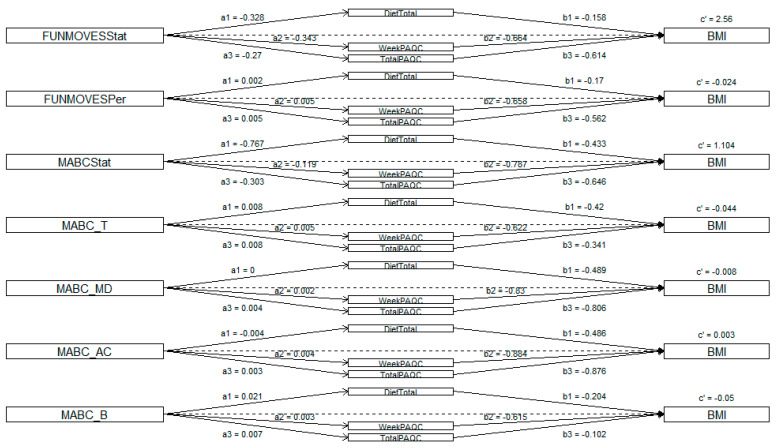
Path coefficients (a′, b′, c′) from mediation models relating motor competence (FUNMOVES status, FUNMOVES percentile, MABC status, MABC total, MABC manual dexterity, MABC aiming and catching, MABC balance) to BMI via: total physical activity, weekly physical activity, and diet.

## Data Availability

The datasets and code generated during the current study are available as Supplementary Materials.

## Supplementary Materials

The following supporting information can be downloaded at https://www.mdpi.com/article/10.3390/children12091178/s1, Database; R Code.

## Abbreviations

The following abbreviations are used in this manuscript:

DCD	Developmental Coordination Disorder
BMI	Body Mass Index
PAQ-C	Physical Activity Questionnaire for Children
APA	American Psychiatric Association
WHO	World Health Organization
MABC-2	Movement Assessment Battery for Children—Second Edition (MABC-2)
PA	Physical Activity

## Appendix A

This appendix presents the BMI classification thresholds used in the study, as defined by each of the four reference systems. These tables provide specific cutoff values for categorizing.

**Table A1:** Diet classification of the participants.

Tool	Category	TD	pDCD
FUNMOVES	Very low quality	6 (2.9%)	1 (3.5%)
	Need improvement	119 (57.2%)	19 (65.5%)
	Optimus diet	83 (39.9%)	9 (31%)
	**Total**	**208**	**29**
MABC-2	Very low quality	0	1 (6.3%)
	Need improvement	23 (69.7%)	10 (62.5%)
	Optimus diet	10 (30.3%)	5 (31.2%)
	**Total**	**33**	**16**

Note: Values are presented as n (%), where n is the number of participants and % is the percentage within the TD group.

**Table A2:** Physical activity classification of the participants based on the total PAQ-C score.

Tool	Category	TD	pDCD
FUNMOVES	Very low	15 (8.1%)	6 (23.1%)
	Low	92 (49.7%)	14 (53.8%)
	Regular	68 (36.8%)	6 (23.1%)
	Intense	10 (5.4%)	0
	**Total**	**185**	**26**
MABC-2	Very low	5 (16.1%)	5 (38.5%)
	Low	14 (45.2%)	7 (53.8%)
	Regular	12 (38.7%)	1 (7.7%)
	Intense	0	0
	**Total**	**31**	**13**

Note: Values are presented as n (%), where n is the number of participants and % is the percentage within the TD group.

**Table A3:** Physical activity classification of the participants based on the weekly PAQ-C score.

Tool	Category	TD	pDCD
FUNMOVES	Very low	8 (4.3%)	4 (15.4%)
	Low	53 (28.6%)	11 (42.3%)
	Regular	93 (50.3%)	10 (38.5%)
	Intense	31 (16.8%)	1 (3.8%)
	**Total**	**185**	**26**
MABC-2	Very low	3 (9.7%)	2 (15.4%)
	Low	13 (41.9%)	6 (46.2%)
	Regular	10 (32.3%)	5 (38.4%)
	Intense	5 (16.1%)	0
	**Total**	**31**	**13**

Note: Values are presented as n (%), where n is the number of participants and % is the percentage within the TD group.

**Table A4:** Physical activity classification of the participants based on the raw scores (PAQ-C).

Tool	Category	TD	pDCD
FUNMOVES	Total score: mean (SD)	2.87 (0.63)	2.54 (0.69)
	Weekly score: mean (SD)	3.20 (0.74)	2.77 (0.77)
MABC-2	Total score: mean (SD)	2.71 (0.69)	2.26 (0.57)
	Weekly score: mean (SD)	2.94 (0.80)	2.68 (0.75)

**Table A5:** Body fat percentage descriptive statistics for TD and pDCD participants.

Tool	Category	TD	pDCD
FUNMOVES	Body fat: mean (SD)	22.06 (5.81)	28.9 (8.69)
MABC-2	Body fat: mean (SD)	24.82 (6.40)	28.46 (10.99)

## Appendix B

This appendix presents the BMI classification thresholds used in the study, as defined by each of the four reference systems. These tables provide the specific cutoff values for categorizing participants into underweight, normal weight, overweight, or obese, according to age and sex, where applicable. The reference systems included are Orbegozo Foundation (Table A1), Spanish Pediatric Association (Asociación Española de Pediatría—AEP) (Table A2), World Health Organization—WHO (Table A3), and the classification proposed by Arriba (Table A4). These thresholds served as the basis for the composite classification described in Section 2.

**Table A6:** Orbegozo Foundation classification.

	Boys			Girls		
Age (Years)	UW P4	OW P79	Ob P97.5	UW P4	OW P79	Ob P97.5
6	14	17.4	20.1	14.1	18.1	20.9
6.5	14	17.6	20.5	14.1	18.4	21.5
7	14	17.8	21	14.3	18.9	22.4
7.5	14.1	18.1	21.6	14.4	19.5	23.4
8	14.2	18.6	22.4	14.6	20	24.1
8.5	14.4	19.1	23.5	14.8	20.5	24.7
9	14.6	19.8	24.6	14.9	20.9	25.2
9.5	14.8	20.2	25.3	15.1	21.2	25.6
10	14.9	20.4	25.6	15.3	21.6	26.1
10.5	14.9	20.7	26	15.5	21.9	26.4
11	15.1	20.9	26.3	15.6	22.1	26.6
11.5	15.3	21.2	26.7	15.7	22.3	26.8
12	15.5	21.5	27	15.9	22.5	27
12.2	15.7	21.8	27.3	16.1	22.8	27.4

Note: UW—underweight, OW—overweight, Ob—obesity. Percentile thresholds.

**Table A7:** AEP classification.

	Boys			Girls		
Age (Years)	UW −2sd	OW +1sd	Ob +2sd	UW −2sd	OW +1sd	Ob +2sd
6	12.9	17.4	18.9	13	17.2	18.6
6.5	12.9	17.4	18.9	13	17.2	18.6
7	13	17.8	19.4	12.8	17.9	19.6
7.5	13	17.8	19.4	12.8	17.9	19.6
8	13.1	18.5	20.3	12.9	18.6	20.5
8.5	13.1	18.5	20.3	12.9	18.6	20.5
9	13.3	19.3	21.3	13	19.3	21.4
9.5	13.3	19.3	21.3	13	19.3	21.4
10	13.5	20.1	22.3	13.3	19.9	22.1
10.5	13.5	20.1	22.3	13.3	19.9	22.1
11	13.6	20.8	23.2	13.4	20.6	23
11.5	13.6	20.8	23.2	13.4	20.6	23
12	13.9	21.4	23.9	13.9	21.1	23.5
12.2	13.9	21.4	23.9	13.9	21.1	23.5

Note: UW—underweight, OW—overweight, Ob—obesity, SD—deviation thresholds.

**Table A8:** WHO classification.

	Boys			Girls		
Age (Years)	UW −2sd	OW +1sd	Ob +2sd	UW −2sd	OW +1sd	Ob +2sd
6	12.9	16.8	18.5	12.60	17.00	19.20
6.5	13	16.9	18.7	12.60	17.10	19.50
7	13	17	19	12.60	17.30	19.80
7.5	13.1	17.2	19.3	12.70	17.50	20.10
8	13.2	17.4	19.7	12.80	17.70	20.60
8.5	13.3	17.7	20.1	12.90	18.00	21.00
9	13.4	17.9	20.5	13.00	18.30	21.50
9.5	13.5	18.2	20.9	13.20	18.70	22.00
10	13.6	18.5	21.4	13.40	19.00	22.60
10.5	13.8	18.8	21.9	13.60	19.40	23.10
11	14	19.2	22.5	13.80	19.90	23.70
11.5	14.1	19.5	23	14.00	20.30	24.30
12	14.4	19.9	23.6	14.30	20.80	25.00
12.2	14.6	20.4	24.2	14.60	21.30	25.60

Note: UW—underweight, OW—overweight, Ob—obesity, SD—deviation thresholds.

**Table A9:** Arriba classification.

	Boys			Girls		
Age (Years)	UW −2sd	OW +1sd	Ob +2sd	UW −2sd	OW +1sd	Ob +2sd
6	12.6	17.4	19	12.5	17.6	19.3
6.5	12.6	17.4	19	12.5	17.6	19.3
7	12.5	18.2	20.1	12.4	18.4	20.4
7.5	12.5	18.2	20.1	12.4	18.4	20.4
8	12.5	19.1	21.3	12.7	19.3	21.5
8.5	12.5	19.1	21.3	12.7	19.3	21.5
9	12.9	19.8	22.1	12.8	19.7	22
9.5	12.9	19.8	22.1	12.8	19.7	22
10	13.2	20.7	23.2	12.9	20.7	23.3
10.5	13.2	20.7	23.2	12.9	20.7	23.3
11	13.1	21.2	23.9	13.2	21.3	24
11.5	13.1	21.2	23.9	13.2	21.3	24
12	13.2	21.9	24.8	13.3	22.6	25.7
12.2	13.2	21.9	24.8	13.3	22.6	25.7

Note: UW—underweight, OW—overweight, Ob—obesity, SD—deviation thresholds.

## Appendix C

This appendix includes the full version of the Krece Plus questionnaire used to assess dietary habits in children and adolescents. The instrument provides a total score and a categorical classification of dietary quality (poor, needs improvement, optimal). Although the questionnaire also includes items related to physical activity, only the dietary component was used in this study. The validated Spanish version is presented below to facilitate replication and comparison in future research. Additionally, an English translation of the questionnaire is provided in this appendix solely for comprehension purposes; this version has been translated exclusively for reference and does not constitute a validated instrument.

Spanish
Señale si/no en las siguientes afirmaciones con respecto a su hijo/a

**Afirmación**	**Sí**	**No**
1. Toma una fruta o zumo natural todos los días		
2. Toma una 2º pieza de fruta todos los días		
3. Toma verduras frescas (ensaladas) o cocinadas regularmente una vez al día		
4. Toma verduras frescas o cocinadas de forma regular más de una vez al día		
5. Consume pescado con regularidad (por lo menos 2–3 veces por semana)		
6. Acude una vez o más a la semana a un centro de comida rápida (Fast food) tipo hamburguesería		
7. Le gustan las legumbres y las toma más de 1 vez a la semana		
8. Toma pasta o arroz casi a diario (5 días o más a la semana)		
9. Desayuna un cereal o derivado (pan, etc)		
10.Toma frutos secos con regularidad (al menos 2–3 veces a la semana)		
11. Se utiliza aceite de oliva en casa		
12. No desayuna		
13. Desayuna un lácteo (yogurt, leche, etc)		
14. Desayuna bollería industrial, galletas o pastelitos.		
15. Toma 2 yogures y/o 40 g de queso cada día		
16. Toma golosinas y/o caramelos varias veces al día		

¿Cuántas horas ves la televisión o juegas a videojuegos diariamente de promedio?	0	1	2	3	4	5
¿Cuántas horas dedicas a actividades extraescolares semanalmente?	0	1	2	3	4	5

English
Please indicate Yes/No for the following statements:

**Statement**	**Yes**	**No**
1. Eats a fruit or natural juice every day		
2. Eats a second piece of fruit every day		
3. Eats fresh (salads) or cooked vegetables regularly once a day		
4. Eats fresh or cooked vegetables regularly more than once a day		
5. Eats fish regularly (at least 2–3 times per week)		
6. Goes to a fast-food restaurant (e.g., burger place) once a week or more		
7. Likes legumes and eats them more than once a week		
8. Eats pasta or rice almost daily (5 days or more per week)		
9. Has cereal or cereal-based products (bread, etc.) for breakfast		
10. Eats nuts regularly (at least 2–3 times per week)		
11. Olive oil is used at home		
12. Does not eat breakfast		
13. Eats a dairy product (yogurt, milk, etc.) for breakfast		
14. Eats industrial pastries, cookies, or small cakes for breakfast		
15. Eats 2 yogurts and/or 40 g of cheese daily		
16. Eats candies and/or sweets several times a day		

How many hours do he/she watch TV or play video games daily on average?	0	1	2	3	4	5
How many hours per week do he/she spend on extracurricular activities?	0	1	2	3	4	5

## Appendix D

This appendix presents the complete Physical Activity Questionnaire for Children (PAQ-C), a validated tool designed to measure general physical activity levels over the previous week in children. The questionnaire includes multiple items assessing different contexts of activity, as well as a specific item on typical weekly activity (Item 9). The Spanish version used in this study is provided below to support reproducibility and allow for consistent application in similar populations. Although a validated English version of the PAQ-C does exist, the translation included in this appendix has been prepared solely for comprehension purposes and should not be considered the validated instrument.

Spanish

1 Actividad Física en tu tiempo libre: ¿Has hecho alguna de estas actividades en los últimos 7 días (última semana)? Si tu respuesta es sí: ¿cuántas veces las has hecho? (Marca un solo cícrulo por actividad)

	NO	1–2	3–4	5–6	7 veces o +
Saltar a la comba	❍	❍	❍	❍	❍
Patinar	❍	❍	❍	❍	❍
Jugar a juegos como el pilla-pilla	❍	❍	❍	❍	❍
Montar en bicicleta	❍	❍	❍	❍	❍
Caminar (como ejercicio)	❍	❍	❍	❍	❍
Correr/footing	❍	❍	❍	❍	❍
Aeróbic/spinning	❍	❍	❍	❍	❍
Natación	❍	❍	❍	❍	❍
Bailar/danza	❍	❍	❍	❍	❍
Bádminton	❍	❍	❍	❍	❍
Rugby	❍	❍	❍	❍	❍
Montar en monopatín	❍	❍	❍	❍	❍
Fútbol/fútbol sala	❍	❍	❍	❍	❍
Voleibol	❍	❍	❍	❍	❍
Hockey	❍	❍	❍	❍	❍
Baloncesto	❍	❍	❍	❍	❍
Esquiar	❍	❍	❍	❍	❍
Otros deportes de raqueta	❍	❍	❍	❍	❍
Balonmano	❍	❍	❍	❍	❍
Atletismo	❍	❍	❍	❍	❍
Musculación/pesas	❍	❍	❍	❍	❍
Artes marciales (judo, kárate, etc.)	❍	❍	❍	❍	❍
Otros:	❍	❍	❍	❍	❍
Otros:	❍	❍	❍	❍	❍

2 En los últimos 7 días, durante las clases de educación física, ¿cuántas veces estuviste muy activo/a durante las clases: jugando intensamente, corriendo, saltando, haciendo lanzamientos? (Señala sólo una)

No hice/hago Educación Física	❍
Casi nunca	❍
Algunas veces	❍
A menudo	❍
Siempre	❍

3 En los últimos 7 días, ¿qué hiciste en el tiempo de descanso? (Señala sólo una)

Estar sentado/a (hablar, leer, trabajo de clase, etc.)	❍
Estar o pasear por los alrededores	❍
Correr o jugar un poco	❍
Correr y jugar bastante	❍
Correr y jugar intensamente todo el tiempo	❍

4 En los últimos 7 días, ¿qué hiciste normalmente a la hora de la comida (antes y después de comer)? (Señala sólo una)

Estar sentado/a (hablar, leer, trabajo de clase, etc.)	❍
Estar o pasear por los alrededores	❍
Correr o jugar un poco	❍
Correr y jugar bastante	❍
Correr y jugar intensamente todo el tiempo	❍

5 En los últimos 7 días, inmediatamente después del colegio, hasta las 6 h, ¿cuántos días jugaste a algún juego, hiciste deporte o bailes en los que estuvieras muy activo/a? (Señala sólo una)

Ninguno	❍
1 vez en la última semana	❍
2–3 veces en la última semana	❍
4 veces en la última semana	❍
5 veces o más en la última semana	❍

6 En los últimos 7 días, cuantos días a partir de media tarde (entre las 6 h y las 10 h) hiciste deportes, baile o jugaste a juegos en los que estuvieras muy activo/a? (Señala sólo una)

Ninguno	❍
1 vez en la última semana	❍
2–3 veces en la última semana	❍
4–5 veces en la última semana	❍
6–7 veces o más en la última semana	❍

7 El último fin de semana, ¿cuántas veces hiciste deportes, baile o jugar a juegos en los que estuviste muy activo/a? (Señala sólo una)

Ninguno	❍
1 vez en la última semana	❍
2–3 veces en la última semana	❍
4–5 veces en la última semana	❍
6 o más veces o más en la última semana	❍

8 ¿Cuál de las siguientes frases describen mejor tu última semana? Lee las cinco antes de decidir cuál te describe mejor. (Señala sólo una)

Todo o la mayoría de mi tiempo libre lo dediqué a actividades que suponen poco esfuerzo físico	❍
Algunas veces (1 o 2 veces) hice actividades físicas en mi tiempo libre (por ejemplo, hacer deportes, correr, nadar, montar en bicicleta, hacer aeróbic)	❍
A menudo (3–4 veces a la semana) hice actividad física en mi tiempo libre	❍
Bastante a menudo (5–6 veces en la última semana) hice actividad física en mi tiempo libre	❍
Muy a menudo (7 o más veces en la última semana) hice actividad física en mi tiempo libre	❍

9 Señala con qué frecuencia hiciste actividad física cada día de la semana pasada (como hacer deporte, jugar, bailar o cualquier otra actividad física)

	**Ninguna**	**Poca**	**Normal**	**Bastante**	**Mucha**
Lunes	❍	❍	❍	❍	❍
Martes	❍	❍	❍	❍	❍
Miércoles	❍	❍	❍	❍	❍
Jueves	❍	❍	❍	❍	❍
Viernes	❍	❍	❍	❍	❍
Sábado	❍	❍	❍	❍	❍
Domingo	❍	❍	❍	❍	❍

10 ¿Estuviste enfermo/a esta última semana o algo impidió que hicieras normalmente actividades físicas?

Sí ❍

No ❍

Si la respuesta es sí, ¿qué lo impidió? ………………………………………………

English

1 Physical activity in your spare time: Have you done any of the following activities in the past 7 days (last week)? If yes, how many times? (Mark only one circle per row.)

	NO	1–2	3–4	5–6	7 veces o +
Skipping	❍	❍	❍	❍	❍
Rowing	❍	❍	❍	❍	❍
Playing physical active games	❍	❍	❍	❍	❍
Bicycling	❍	❍	❍	❍	❍
Walking for exercise	❍	❍	❍	❍	❍
Jogging or running	❍	❍	❍	❍	❍
Aerobics/spinning	❍	❍	❍	❍	❍
Swimming	❍	❍	❍	❍	❍
Dance	❍	❍	❍	❍	❍
Badminton	❍	❍	❍	❍	❍
Rugby	❍	❍	❍	❍	❍
Riding	❍	❍	❍	❍	❍
Soccer/Soccer indoor	❍	❍	❍	❍	❍
Volleyball	❍	❍	❍	❍	❍
Hockey	❍	❍	❍	❍	❍
Basketball	❍	❍	❍	❍	❍
Ski	❍	❍	❍	❍	❍
Tennis or similar	❍	❍	❍	❍	❍
Handball	❍	❍	❍	❍	❍
Athletics	❍	❍	❍	❍	❍
Gym work out	❍	❍	❍	❍	❍
Martial arts	❍	❍	❍	❍	❍
Otros:	❍	❍	❍	❍	❍
Otros:	❍	❍	❍	❍	❍

2 In the last 7 days, during your physical education (PE) classes, how often were you very active (playing hard, running, jumping, throwing)? (Check one only.)

I don’t do PE	❍
Hardly ever	❍
Sometimes	❍
Quite often	❍
Always	❍

3 In the last 7 days, what did you do most of the time at recess? (Check one only.)

Sat down (talking, reading, doing schoolwork)	❍
Stood around or walked around	❍
Ran or played a little bit	❍
Ran around and played quite a bit	❍
Ran and played hard most of the time	❍

4 In the last 7 days, what did you normally do at lunch (besides eating lunch)? (Check one only.)

Sat down (talking, reading, doing schoolwork)	❍
Stood around or walked around	❍
Ran or played a little bit	❍
Ran around and played quite a bit	❍
Ran and played hard most of the time	❍

5 In the last 7 days, on how many days right after school, did you do sports, dance, or play games in which you were very active? (Check one only.)

None	❍
1 time last week	❍
2–3 times last week	❍
4 times last week	❍
5 times or more last week	❍

6 In the last 7 days, on how many evenings (18.00–22.00h) did you do sports, dance, or play games in which you were very active? (Check one only.)

None	❍
1 time last week	❍
2–3 times last week	❍
4–5 times last week	❍
6–7 times last week	❍

7 On the last weekend, how many times did you do sports, dance, or play games in which you were very active? (Check one only.)

None	❍
1 time last week	❍
2–3 times last week	❍
4–5 times last week	❍
6 or more times last week	❍

8 Which one of the following describes you best for the last 7 days? Read all five statements before deciding on the one answer that describes you)

All or most of my free time was spent doing things that involve little physical effort	❍
All or most of my free time was spent doing things that involve little physical effort	❍
I often (3–4 times last week) did physical things in my free time	❍
I quite often (5–6 times last week) did physical things in my free time	❍
I very often (7 or more times last week) did physical things in my free time	❍

9 Mark how often you did physical activity (like playing sports, games, doing dance, or any other physical activity) for each day last week.

	**None**	**Little Bit**	**Medium**	**Often**	**Very Often**
Monday	❍	❍	❍	❍	❍
Tuesday	❍	❍	❍	❍	❍
Wednesday	❍	❍	❍	❍	❍
Thursday	❍	❍	❍	❍	❍
Friday	❍	❍	❍	❍	❍
Saturday	❍	❍	❍	❍	❍
Sunday	❍	❍	❍	❍	❍

10 Were you sick last week, or did anything prevent you from doing your normal physical activities? (Check one.)

Yes ❍

No ❍

If Yes, what prevented you? ………………………………………………

## Appendix E

This table summarizes mediation models assessing the relationship between motor competence (predictors), health-related behaviors (mediators), and BMI (criterion). Each model reports total, direct, and indirect effects with 95% confidence intervals. The final column indicates the mediation type “Direct only” (significant direct effect, no mediation), “Partial mediation” (significant indirect and direct effects), or “No effect” (no significant pathways).

**Table A10:** Mediation models.

Predictive Variable	Mediation Variable	Criterion	Total Effect	CI (95%)	Direct Effect	CI (95%)	Indirect Effect	CI (95%)	Result Effects
FUNMOVESStat	DietTotal	BMI	2.61 *	[0.95, 4.34]	2.56 *	[0.93, 4.27]	0.05	[−0.08, 0.27]	Direct only
FUNMOVESStat	WeekPAQC	BMI	2.61 *	[0.90, 4.37]	2.38 *	[0.69, 4.14]	0.23 *	[−0.004, 0.53]	Partial mediation
FUNMOVESStat	TotalPAQC	BMI	2.61 *	[0.96, 4.45]	2.45 *	[0.80, 4.27]	0.17*	[−0.02, 0.42]	Partial mediation
FUNMOVESPer	DietTotal	BMI	−0.025 *	[−0.039, −0.011]	−0.024 *	[−0.038, −0.010]	0.0003	[−0.0027, 0.0014]	Direct only
FUNMOVESPer	WeekPAQC	BMI	−0.025 *	[−0.039, −0.011]	−0.021 *	[−0.036, −0.007]	−0.0032 *	[−0.0071, −0.0005]	Partial mediation
FUNMOVESPer	TotalPAQC	BMI	−0.025 *	[−0.039, −0.011]	−0.0218 *	[−0.0360, −0.0085]	−0.0027 *	[−0.0065, −0.0001]	Partial mediation
MABCStat	DietTotal	BMI	1.43	[−1.47, 4.43]	1.10	[−1.74, 4.05]	0.33	[−0.38, 1.40]	No effect
MABCStat	WeekPAQC	BMI	1.43	[−1.50, 4.51]	1.34	[−1.55, 4.48]	0.09	[−0.55, 0.49]	No effect
MABCStat	TotalPAQC	BMI	1.43	[−1.49, 4.33]	1.24	[−1.72, 4.26]	0.20	[−0.45, 0.81]	No effect
MABC_T	DietTotal	BMI	−0.047 *	[−0.087, −0.006]	−0.0438 *	[−0.0836, −0.002]	−0.0035	[−0.020, 0.008]	Direct only
MABC_T	WeekPAQC	BMI	−0.047 *	[−0.088, −0.007]	−0.0442 *	[−0.0873, −0.003]	−0.0031	[−0.0119, 0.0072]	Direct only
MABC_T	TotalPAQC	BMI	−0.047 *	[−0.086, −0.006]	−0.0447 *	[−0.0858, −0.001]	−0.0026	[−0.0169, 0.0138]	Direct only
MABC_MD	DietTotal	BMI	−0.008	[−0.047, 0.037]	−0.0082	[−0.050, 0.035]	0.00008	[−0.010, 0.019]	No effect
MABC_MD	WeekPAQC	BMI	−0.008	[−0.047, 0.036]	−0.0066	[−0.047, 0.036]	−0.0015	[−0.0084, 0.0086]	No effect
MABC_MD	TotalPAQC	BMI	−0.008	[−0.047, 0.038]	−0.0047	[−0.0467, 0.0425]	−0.0034	[−0.0134, 0.0071]	No effect
MABC_AC	DietTotal	BMI	0.005	[−0.040, 0.051]	0.0028	[−0.041, 0.048]	0.0018	[−0.0089, 0.0125]	No effect
MABC_AC	WeekPAQC	BMI	0.005	[−0.042, 0.049]	0.0079	[−0.039, 0.053]	−0.0033	[−0.0145, 0.0024]	No effect
MABC_AC	TotalPAQC	BMI	0.005	[−0.042, 0.051]	0.0070	[−0.0388, 0.0534]	−0.0023	[−0.0129, 0.0034]	No effect
MABC_B	DietTotal	BMI	−0.054 *	[−0.093, −0.016]	−0.0495 *	[−0.088, −0.013]	−0.0044	[−0.0226, 0.0103]	Direct only
MABC_B	WeekPAQC	BMI	−0.054 *	[−0.093, −0.016]	−0.0520 *	[−0.0936, −0.013]	−0.0019	[−0.0078, 0.0084]	Direct only
MABC_B	TotalPAQC	BMI	−0.054 *	[−0.091, −0.016]	−0.0532 *	[−0.0935, −0.014]	−0.0007	[−0.0102, 0.0166]	Direct only

Note: FUNMOVESStat, status by FUNMOVES (1 = pDCD); FUNMOVESPer, percentile by FUNMOVES; MABCStat, status by FUNMOVES (1 = pDCD); MABC_T, percentile by MABC total score; MABC_MD, percentile by MABC manual dexterity; MABC_AC, percentile by MABC aiming and catching; MABC_B, percentile by MABC balance. * *p* < 0.05.

## Appendix F

This appendix also provides the completed STROBE (Strengthening the Reporting of Observational Studies in Epidemiology) checklist. The inclusion of this checklist ensures adherence to internationally recognized reporting standards for observational studies, thereby enhancing the transparency, rigor, and reproducibility of the research.

	**Item No.**	**Recommendation**
**Title** **and abstract**	1	(a) Indicate the study’s design with a commonly used term in the title or the abstract
		(b) Provide in the abstract an informative and balanced summary of what was performed and what was found
**Introduction**		
Background/rationale	2	Explain the scientific background and rationale for the investigation being reported
Objectives	3	State specific objectives, including any pre-specified hypotheses
**Methods**		
Study design	4	Present key elements of the study design early in the paper
Setting	5	Describe the setting, locations, and relevant dates, including periods of recruitment, exposure, follow-up, and data collection
Participants	6	Give the eligibility criteria and the sources and methods of the selection of participants
Variables	7	Clearly define all outcomes, exposures, predictors, potential confounders, and effect modifiers. Give diagnostic criteria, if applicable
Data sources/measurement	8	For each variable of interest, give sources of data and details of methods of assessment (measurement). Describe the comparability of assessment methods if there is more than one group
Bias	9	Describe any efforts to address potential sources of bias
Study size	10	Explain how the study size was arrived at
Quantitative variables	11	Explain how quantitative variables were handled in the analyses. If applicable, describe which groupings were chosen and why
Statistical methods	12	(a) Describe all statistical methods, including those used to control for confounding
		(b) Describe any methods used to examine subgroups and interactions
		(c) Explain how missing data were addressed
		(d) If applicable, describe analytical methods taking account of the sampling strategy
		(e) Describe any sensitivity analyses
**Results**		
Participants	13	(a) Report numbers of individuals at each stage of study—e.g., numbers potentially eligible, examined for eligibility, confirmed eligible, included in the study, completing follow-up, and analyzed
		(b) Give reasons for non-participation at each stage
		(c) Consider the use of a flow diagram
Descriptive data	14*	(a) Give characteristics of study participants (e.g., demographic, clinical, social) and information on exposures and potential confounders
		(b) Indicate the number of participants with missing data for each variable of interest
Outcome data	15*	Report numbers of outcome events or summary measures
Main results	16	(a) Give unadjusted estimates and, if applicable, confounder-adjusted estimates and their precision (e.g., 95% confidence interval). Make clear which confounders were adjusted for and why they were included
		(b) Report category boundaries when continuous variables were categorized
		(c) If relevant, consider translating estimates of relative risk into absolute risk for a meaningful time period
Other analyses	17	Report other analyses performed—e.g., analyses of subgroups and interactions and sensitivity analyses
**Discussion**		
Key results	18	Summarize key results with reference to study objectives
Limitations	19	Discuss limitations of the study, taking into account sources of potential bias or imprecision. Discuss both the direction and magnitude of any potential bias
Interpretation	20	Give a cautious overall interpretation of results, considering objectives, limitations, multiplicity of analyses, results from similar studies, and other relevant evidence
Generalizability	21	Discuss the generalizability (external validity) of the study results
**Other information**		
Funding	22	Give the source of funding and the role of the funders for the present study and, if applicable, for the original study on which the present article is based
